# [^18^F]mFBG PET-CT for detection and localisation of neuroblastoma: a prospective pilot study

**DOI:** 10.1007/s00259-022-06063-6

**Published:** 2022-12-12

**Authors:** Atia Samim, Thomas Blom, Alex J. Poot, Albert D. Windhorst, Marta Fiocco, Nelleke Tolboom, Arthur J. A. T. Braat, Sebastiaan L. Meyer Viol, Rob van Rooij, Max M. van Noesel, Marnix G. E. H. Lam, Godelieve A. M. Tytgat, Bart de Keizer

**Affiliations:** 1 Princess Máxima Centre for Paediatric Oncology, Heidelberglaan 25, 3584 CS Utrecht, Netherlands; 2 Division Imaging & Oncology, University Medical Centre Utrecht, Utrecht, Netherlands; 3 Department of Radiology and Nuclear Medicine, Cancer Centre Amsterdam, Amsterdam University Medical Centres, Amsterdam, Netherlands; 4 Mathematical Institute, Leiden University, Leiden, Netherlands

**Keywords:** Neuroblastoma, Paediatric oncology, Nuclear medicine imaging, [^123^I]mIBG, [^18^F]mFBG, PET-CT

## Abstract

**Purpose:**

*Meta*-[^18^F]fluorobenzylguanidine ([^18^F]mFBG) is a positron emission tomography (PET) radiotracer that allows for fast and high-resolution imaging of tumours expressing the norepinephrine transporter. This pilot study investigates the feasibility of [^18^F]mFBG PET-CT for imaging in neuroblastoma.

**Methods:**

In a prospective, single-centre study, we recruited children with neuroblastoma, referred for *meta*-[^123^I]iodobenzylguanidine ([^123^I]mIBG) scanning, consisting of total body planar scintigraphy in combination with single-photon emission computed tomography-CT (SPECT-CT). Within two weeks of [^123^I]mIBG scanning, total body PET-CTs were performed at 1 h and 2 h after injection of [^18^F]mFBG (2 MBq/kg). Detected tumour localisations on scan pairs were compared. Soft tissue disease was quantified by number of lesions and skeletal disease by SIOPEN score.

**Results:**

Twenty paired [^123^I]mIBG and [^18^F]mFBG scans were performed in 14 patients (median age 4.9 years, *n* = 13 stage 4 disease and *n* = 1 stage 4S). [^18^F]mFBG injection was well tolerated and no related adverse events occurred in any of the patients. Mean scan time for [^18^F]mFBG PET-CT (9.0 min, SD 1.9) was significantly shorter than for [^123^I]mIBG scanning (84.5 min, SD 10.5), *p* < 0.01. Most tumour localisations were detected on the 1 h versus 2 h post-injection [^18^F]mFBG PET-CT. Compared to [^123^I]mIBG scanning, [^18^F]mFBG PET-CT detected a higher, equal, and lower number of soft tissue lesions in 40%, 55%, and 5% of scan pairs, respectively, and a higher, equal, and lower SIOPEN score in 55%, 30%, and 15% of scan pairs, respectively. On average, two more soft tissue lesions and a 6-point higher SIOPEN score were detected per patient on [^18^F]mFBG PET-CT compared to [^123^I]mIBG scanning.

**Conclusion:**

Results of this study demonstrate feasibility of [^18^F]mFBG PET-CT for neuroblastoma imaging. More neuroblastoma localisations were detected on [^18^F]mFBG PET-CT compared to [^123^I]mIBG scanning. [^18^F]mFBG PET-CT shows promise for future staging and response assessment in neuroblastoma.

**Trial registration:**

Dutch Trial Register NL8152.

**Supplementary Information:**

The online version contains supplementary material available at 10.1007/s00259-022-06063-6.

## Introduction

Neuroblastoma is a tumour that originates from the neural crest cells of the sympathetic nervous system. It is the most common extracranial solid malignancy in children, and 90% of patients are younger than 5 years of age at diagnosis [1, 2]. Over 50% of patients present with distant skeletal and/or soft tissue metastases, which is an important prognostic factor for a poor outcome with a long-term survival of only 50% [3, 4].

Nuclear medicine imaging plays an essential role in detecting neuroblastoma localisations. Currently, *meta*-[^123^I]iodobenzylguanidine ([^123^I]mIBG) scanning is first-line for staging, response assessment, follow-up of neuroblastoma, and selection of eligible patients for [^131^I]mIBG therapy [3, 5, 6]. [^123^I]mIBG is a norepinephrine analogue that is radiolabelled with gamma-emitting iodine-123 (^123^I, half-life 13 h). Proper patient preparation with medication is required to protect the thyroid from radioactive iodide [7, 8]. [^123^I]mIBG is taken up by cells via the norepinephrine transporter (NET), and 24 h post-injection, gamma emission of [^123^I]mIBG is visualized with (2D/planar) total body scintigraphy and 3D single-photon emission CT combined with CT (SPECT-CT) of a limited part of the body [9–11]. Due to the long scan time and young age of these patients, procedural sedation during scanning is often necessary.

Compared to scintigraphy/SPECT-CT, positron emission tomography (PET) provides a shorter scan time, higher resolution images, and 3D PET-CT of the total body, which could improve detection and localization of neuroblastoma lesions. Furthermore, PET is more suitable for quantifying radiotracer uptake [12]. In recent years, several PET radiotracers with different molecular targets have been introduced for neuroblastoma imaging, such as [^124^I]mIBG, [^18^F]FDG, [^18^F]F-DOPA, and [^68^ Ga]Ga-DOTA-peptides, generally showing more lesions on PET than paired [^123^I]mIBG scans [13–17]. The EANM guideline on nuclear medicine imaging in neuroblastoma recommends [^18^F]FDG, [^18^F]F-DOPA, and [^68^ Ga]Ga-DOTA-peptides as second-line imaging, of which [^18^F]FDG has limited specificity for skeletal disease due to high physiological bone marrow uptake after therapy [5].

*Meta*-[^18^F]fluorobenzylguanidine ([^18^F]mFBG) is radiolabelled with positron-emitting fluorine-18 (^18^F, half-life 110 min) instead of ^123^I. [^18^F]mFBG stands out as PET tracer because of its uptake via the same NET transporter, same-day injection and scanning, and no need for thyroid-protecting medication [18–21]. Recent developments have facilitated automated synthesis of [^18^F]mFBG, and few reports have described the use of [^18^F]mFBG PET-CT in different NET-expressing tumours [22–28]. In one clinical study in five patients with metastasized neuroblastoma, [^18^F]mFBG showed an overall similar (physiological and pathological) distribution to [^123^I]mIBG, however, with detection of additional tumour lesions on [^18^F]mFBG PET-CT [23]. [^18^F]mFBG PET-CT acquisition at 1 h or 2 h post-injection was proposed for optimal tumour-to-background contrast. Due to limited experience on [^18^F]mFBG PET-CT in neuroblastoma, diagnostic value, safety, radiation absorbed dose, and optimal timing for acquisition of [^18^F]mFBG PET-CT have yet to be established.

The aim of this pilot study was to investigate the feasibility of neuroblastoma imaging using [^18^F]mFBG PET-CT in children, by performing 20 paired [^18^F]mFBG and [^123^I]mIBG scans.

## Material and methods

### Study design and participants

This prospective, cross-sectional study was performed at the Princess Máxima Centre for Paediatric Oncology (Utrecht, Netherlands) and approved by the Local Ethics Committee (details can be found in the “Statements and Declaration” section). We recruited paediatric patients with suspected or confirmed neuroblastoma who were referred for a [^123^I]mIBG scan as part of regular clinical care (staging at diagnosis or any response assessment). If parents/care takers or patients had given written informed consent, [^18^F]mFBG PET-CT was performed within a maximally two-week interval before or after the [^123^I]mIBG scan. Exclusion criteria were age > 18 years, pregnancy, and/or poor clinical condition. Patients were eligible for a second paired [^123^I]mIBG and [^18^F]mFBG scan at a later response assessment.

### Procedures [^123^I]mIBG scanning

Patients were prescribed oral thyroid protecting medication (thyroxine, thiamazole, and potassium iodide, according to our national protocol) for three days, starting one day before [^123^I]mIBG injection [8]. Medication known to interfere with [^123^I]mIBG uptake was avoided [7]. Scintigraphy and SPECT-CT were obtained 24 h after intravenous injection of [^123^I]mIBG (4 MBq/kg bodyweight) on a Symbia Intevo 16 Bold SPECT scanner (Siemens Healthineers, Erlangen, Germany). Planar (anterior and posterior) total body scintigraphy was acquired with a 256 × 1024 matrix size (2.4 × 2.4 mm^2^), low-medium-energy (LME) collimators, and 5 cm/min scan speed. SPECT of an area of interest (axial field-of-view of 38.7 cm) was acquired with a 256 × 256 matrix size (2.4 × 2.4 mm^2^); LME collimators; a 15% wide photo peak window centred at 159 keV and similarly sized upper and lower scatter windows; 30 s acquisition time per view; 60 views per head (120 projections in total) with a 3˚ angular step (continuous acquisition); and a non-circular orbit. A low-dose CT scan was acquired using 110 kV and 10–40 mAs depending on height and weight of the patient. Images were reconstructed using xSPECT Broad Quant, 20 iterations with no subsets, attenuation correction, triple energy window scatter correction, and 7.5-mm Gaussian filtering.

### [^18^F]mFBG preparation

[^18^F]mFBG was synthesized at the Radionuclide Centre of the Amsterdam University Medical Centres, location VUmc, in compliance with Good Manufacturing Practices guidelines and standards and produced in single batches according to the procedure described by Rotstein et al.^1–4^ Prior to clinical production, the production method was validated in triplicate. In short, the [^18^F]mFBG precursor ((1r,3r,5r,7r)-spiro[adamantane-2,2′-[1,3]dioxane]-4′,6′-dion-[3-((1,2,3,3-tetrakis(tert-butoxylcarbonyl)guanidino-3-iodonium)methyl)]ylide) was added to the dried [^18^F]-fluoride anion in tetrabutylammonium hydrogen carbonate to form Boc-protected [^18^F]mFBG. A solution of hydrochloric acid (6 M) was added, and the crude reaction mixture was purified by a reversed-phase semi-preparative high-performance liquid chromatography. [^18^F]mFBG was eluted with ethanol and diluted with saline, followed by sterile filtration. Final [^18^F]mFBG injection solutions underwent quality control testing before batch release for patient administration.

### Procedures [^18^F]mFBG PET-CT

Patients received an intravenous injection of [^18^F]mFBG (2 MBq/kg bodyweight, with a minimum activity of 20 MBq, equal activity as for [^18^F]FDG in our centre), without any restrictions in food or medication intake. To assess safety and tolerability of [^18^F]mFBG injection, patients were monitored for 3 h post-injection, and patients/parents were consulted by telephone at 72 h post-injection for any adverse events (according to the Common Terminology Criteria for Adverse Events, version 5.0).

Total body PET-CTs were performed on a Biograph Vision 600 PET-CT scanner (Siemens Healthineers), at both 1 h and 2 h post-injection to assess the optimal timing for image acquisition. First, a low-dose CT scan was acquired with an automatic tube voltage selection and current modulation (reference: 100 kV, 20 mAs) using CARE kV and CARE Dose4D (Siemens Healthcare). Total body PET scanning was performed using continuous bed motion, scanning two passes at 1.6 mm/sec for head to pelvis and 3.2 mm/sec for lower limbs. Low-dose CT was used for attenuation correction of PET data. PET images were reconstructed using point spread function and time of flight modelling, four iterations with five subsets, and 4-mm Gaussian filtering. Image reconstruction matrix was 440 × 440 resulting in 1.65 × 1.65 mm pixels. PET images were reconstructed to a slice thickness of 3 mm.

To obtain time-activity curves and to estimate radiation absorbed doses of [^18^F]mFBG, in patients who were able and willing, a 70-min dynamic PET scan was performed. Image acquisition started directly after injection with a 6-min scan of the heart region for pharmacokinetic modelling, followed by a series of 16 total body passes (3 min/pass for the first eight passes and 5 min/pass for the last eight passes) and a low-dose CT scan. Each pass was reconstructed as a separate frame. The last two passes of the dynamic PET scan were combined to reconstruct the 1 h post-injection PET-CT.

### Measurements of [^18^F]mFBG uptake

Levels of [^18^F]mFBG uptake in different tissues were quantified on all 1 h and 2 h post-injection PET images. Regions/volumes of interest were drawn over various normal organs (salivary, lacrimal, thyroid, and adrenal gland(s); left and right liver lobe; heart wall; pancreas; colon; testes; uterus; kidneys; spleen; muscle; blood pool; lung; bone marrow; breasts; brown and subcutaneous fat; brain) and different types of tumour lesions (primary tumour, up to five distant soft tissue metastases, and up to five skeletal metastases). Mean or maximum standardized uptake value (*SUV*_mean/max_) for normal-organ uptake and lesion uptake were calculated, normalized by lean body mass [29].

Dynamic volumes of interest of different tissues were manually delineated on the 16 frames of the dynamic PET scan and two frames of the 2 h post-injection PET scan using PMOD (version 4.2). Time-activity curves for selected background organs and five randomly selected tumour lesions were generated. Organ residence times were calculated and used as input for dosimetry in OLINDA\EXM (version 1.0). Patient organ masses were scaled in proportion to the patient-to-anatomic model total body mass ratio [30]. Radiation-absorbed dose of [^18^F]mFBG for various organs and total body effective dose were calculated [23, 30]. Effectives doses for [^123^I]mIBG were estimated using the EANM paediatric dosage card (2014) [31].

### Lesion detection

Any [^123^I]mIBG or [^18^F]mFBG uptake in bone (marrow) or soft tissue, exceeding surrounding background activity, was regarded as pathological neuroblastoma uptake, and regarded as tumour lesion. Two readers (AJATB and NT) independently scored anonymized scans for presence of any pathological (skeletal and/or soft tissue) lesions. Discrepancies between readers were resolved by a third consensus reader (BDK). All readers were nuclear medicine physicians with > 5 years of experience in paediatric oncology. Planar total body scintigraphy, SPECT-CT, PET-CT, and PET maximum intensity projection (MIP) were interpreted based on visual assessment, while taking morphologic data from CT scans into account. Clinical information or other imaging findings were not available. Paired [^123^I]mIBG, [^18^F]mFBG 1 h, and 2 h post-injection scans were examined at least one month apart to avoid recollection bias. Semiquantitative Society of Paediatric Oncology European Neuroblastoma Network (SIOPEN) scores were used to quantify extent of skeletal disease [32]. The number of soft tissue lesions were counted per patient.

First, SIOPEN scores and number of soft tissue lesions detected on [^18^F]mFBG PET-CT 1 h and 2 h were compared. The PET-CT acquisition time (1 h or 2 h) with on average more lesions was used to compare with [^123^I]mIBG scanning. Paired [^123^I]mIBG scans and [^18^F]mFBG PET-CT were compared at both a patient level and skeletal segment level (using the skeletal segments of the SIOPEN scoring system). If lesions were only detected on one of the paired scans, it was investigated whether lesions correlated with previous localisations of disease on earlier [^123^I]mIBG scans.

### Statistical analysis

Continuous variables are presented as mean with standard deviation (or median with ranges) and categorical variables as frequencies along with percentages. A paired samples *T*-test or a Wilcoxon signed rank test (in case of violation of asymptotic normality) was applied to assess differences between two scan methods. Difference in proportions between two scan methods was tested by an exact McNemar test. Statistical analyses were performed using SPSS (package version 27), assuming significant differences for *p* < 0.05.

## Results

### Patient and scan characteristics

Between July 2020 and June 2021, 14 consecutive patients who were referred for an [^123^I]mIBG scan were recruited for paired [^18^F]mFBG PET-CT (Table 1). All patients (43% male) had metastasized neuroblastoma (stage 4 or 4S) at diagnosis [33]. Age at scanning ranged from 2 months to 16 years. In six patients, a second paired [^123^I]mIBG-[^18^F]mFBG scan was performed at a later response assessment during course of treatment. In total, 20 paired [^123^I]mIBG-[^18^F]mFBG scans were performed at various time points of response assessment.

**Table 1:** Tumour lesion detection on 20 paired [^123^I]mIBG-[^18^F]mFBG scans

Patients	INSS stage	Age, months	Time point of assessment	Time from diagnosis, months	Scan pair	Total SIOPEN score		Number of soft tissue lesions	
						[^123^I]mIBG	[^18^F]mFBG	[^123^I]mIBG	[^18^F]mFBG
1	4	58	Mid-induction	1	1	26	36	1 (PT)	2 (PT and lesion in neuroforamen)
		66	Pre-IT	9	2	0	0	1 (PT remnant)	7 (separate PT remnants)
2	4	60	Pre-IT	16	3	1	13	0	0
		69	Follow-up	25	4	0	5	0	0
3	4	13	Mid-induction	2	5	5	17	1 (PT)	1 (PT)
4	4	200	Relapse therapy I	38	6	8	21	0	0
		206	Relapse therapy II	44	7	5	11	0	0
5	4	145	Pre-IT	10	8	0	0	0	0
6	4	60	Post-induction	4	9	2	1	1 (PT)	0
7	4S	6	Low risk therapy	3	10	0	0	0	1 (liver lesion)
8	4	61	Pre-IT	13	11	1	11	0	0
		65	Mid-IT	18	12	0	0	0	0
9	4	50	Mid-IT I	20	13	1	21	0	0
		55	Mid-IT II	24	14	0	11	0	0
10	4	197	Follow-up	63	15	1	0	0	1 (PT remnant)
11	4	37	Mid-induction I	1	16	41	40	2 (PT and pleural lesion)	4 (PT, pleural lesion, and 2 mediastinal lesions)
		40	Mid-induction II	4	17	12	19	1 (PT)	3 (PT, pleural lesion, and mediastinal lesion)
12	4	2	Diagnosis	0	18	3	24	2 (PT and liver lesion)	8 (PT, liver lesion, and 6 separate abdominal regional lymph nodes)
13	4	10	Mid-induction	6	19	0	0	8 (PT and 7 subcutaneous nodules)	24 (PT and 23 subcutaneous nodules)
14	4	96	Post-IT	58	20	0	0	1 (PT remnant)	1 (PT remnant)
Median (IQR)		59 (19–89)	-	11 (4–24)	-	1 (0–5)	11 (0–21)	0 (0–1)	1 (0–3)

Tumour lesion detection on 20 paired [^123^I]mIBG-[^18^F]mFBG scans performed in 14 patients with neuroblastoma. Lesions were quantified using total SIOPEN score for skeletal lesions and counting the number of soft tissue lesions, for [^123^I]mIBG scintigraphy plus SPECT-CT and [^18^F]mFBG PET-CT at 1 h post-injection. Patient 11 was treated with amlodipine during both [^123^I]mIBG and [^18^F]mFBG scanning (scan pairs 16 and 17), and patient 13 was treated with amlodipine during only the [^18^F]mFBG PET-CT (scan pair 19)

Abbreviations: [^18^F]mFBG = meta-[^18^F]fluorobenzylguanidine, [^123^I]mIBG = meta-[^123^I]iodobenzylguanidine, IT = immunotherapy, INSS = international neuroblastoma staging system, PT = primary tumour

Median time between [^123^I]mIBG and [^18^F]mFBG scanning was 3.5 days (IQR 1–9). Molar activity of [^18^F]mFBG on time of injection was at least 18.5 GBq/µmol with a median of 54 (range, 31–114). Median administrated activity of [^18^F]mFBG was 37 MBq (range, 20–166) with a median administered pharmaceutical dose of 0.16 µg (range, 0.06–0.98). No related adverse events were observed in any of the patients after [^18^F]mFBG injection. PET-CT scanning was well-tolerated with a significantly shorter mean scan time (9.0 min, SD 1.9) compared with scintigraphy plus SPECT-CT (84.5 min, SD 10.5) and significantly lower rate of procedural sedation (2/20 vs. 10/20, respectively), both *p* < 0.01. In one 2-year-old patient (patient 6), only the 1 h post-injection PET-CT was successfully performed without procedural sedation.

### [^18^F]mFBG distribution and dosimetry

**Fig. 1:**
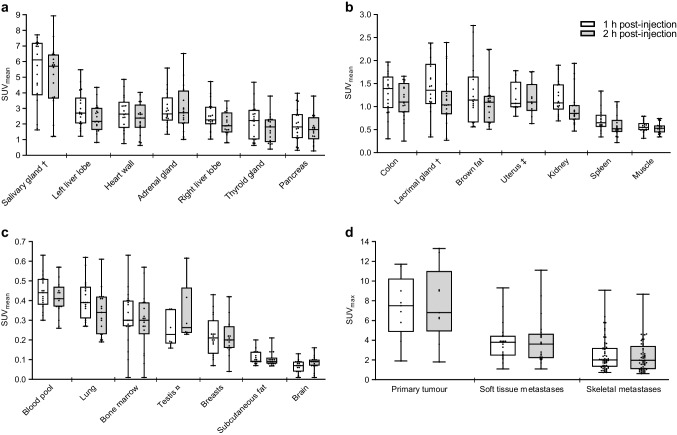
[^18^F]mFBG uptake of normal organs (**a–c**) and tumour lesions (**d**) on 1 h and 2 h post-injection PET-CTs. *SUV*_mean_ of different normal organs (**a–c**) measured on nineteen scans, or if indicated on (†) eighteen scans, (‡) eleven scans, or (¤) six scans. *SUV*_max_ of tumour lesions (**d**) representing nine primary tumour lesions, 21 soft tissue metastases measured on 9 scans, and 60 skeletal metastases measured on 12 scans. Data are presented as median, interquartile range, and range. Each dot represents one measurement. Abbreviations: [^18^F]mFBG, *meta*-[^18^F]fluorobenzylguanidine; *SUV*_mean_ mean standardized uptake value, normalized for lean body mass; *SUV*_max_ maximum standardized uptake value, normalized for lean body mass

[^18^F]mFBG showed prominent activity in the urinary tract, salivary glands, liver, heart wall, adrenal glands, and pancreas at both 1 h and 2 h post-injection (Fig. 1a). Uptake was higher in the left liver lobe compared to the right (1 h post-injection median *SUV*_mean_ of 2.7 [IQR 2.1–3.6] vs. 2.2 [IQR 1.8–2.9], respectively). Regarding uptake in tumour lesions (Fig. 1d), primary tumour lesions showed highest uptake, followed by distant soft tissue metastases, and skeletal metastases (1 h post-injection median SUV_max:_ 7.5 [IQR 7.5–9.0], 3.8 [IQR 2.5–4.5], and 2.1 [IQR 1.4–3.8], respectively).

**Fig. 2:**
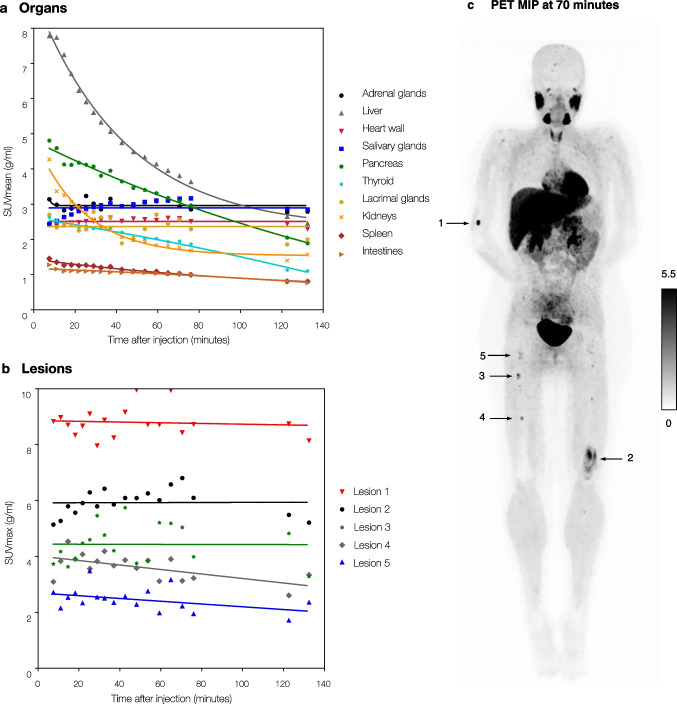
[^18^F]mFBG uptake over time measured by dynamic PET scanning in a 16-year-old female patient. Time-activity curves for normal organs (**a**) and five skeletal lesions (**b**). Each symbol represents the mean or maximum SUV for one PET frame at the mid-time of the corresponding pass (in total 18). **c** Example of a PET maximum intensity projection (MIP) image at 70 min post-injection. Arrows indicate the five randomly chosen skeletal lesions. Earlier and later PET MIP images, including paired [^123^I]mIBG scintigraphy, can be found in the online appendix, Fig. S1. Abbreviations: [^18^F]mFBG = *meta*-[^18^F]fluorobenzylguanidine, [^123^I]mIBG = *meta*-[.^123^I]iodobenzylguanidine, PET = positron emission tomography, SUV = standardized uptake value (normalized for lean body mass)

For most organs (Fig. 1a, 1b, and 1c), uptake levels were slightly lower at 2 h compared to 1 h post-injection. For tumour lesions (Fig. 1d), uptake levels did not differ between 1 and 2 h post-injection. This was also confirmed in time-activity curves, generated from dynamic PET scans that were performed in two patients. Results of one patient are shown in Fig. 2 (a similar pattern was seen for the other patient). Over the first hour after injection, normal organ uptake gradually decreased, after which the curves seemed to flatten out. From 1 to 2 h post-injection, there was a slight decrease in liver, pancreas, and thyroid uptake levels, while uptake levels in other organs and tumour lesions remained more or less stable.

Normal organ radiation absorbed doses for [^18^F]mFBG injection for these two 16-years-old females were comparable (Table 2). Total effective dose of [^18^F]mFBG (3.0 and 3.2 mSv [0.019 and 0.021 mSv/MBq], respectively) was lower than estimated for [^123^I]mIBG (4.6 mSv [0.017 mSv/MBq]).

**Table 2:** [^18^F]mFBG: normal organ absorbed radiation doses

Organ (mGy/MBq)	Dynamic scan 1 (scan 6)	Dynamic scan 2 (scan 15)
Adrenals	0.040	0.020
Brain	0.003	0.003
Breasts	0.007	0.006
Gallbladder wall	0.016	0.016
Lower large intestine wall	0.012	0.013
Small intestine	0.012	0.013
Stomach wall	0.011	0.011
Upper large intestine wall	0.011	0.013
Heart wall	0.047	0.027
Kidneys	0.025	0.016
Liver	0.052	0.046
Lungs	0.014	0.010
Muscle	0.008	0.008
Ovaries	0.013	0.014
Pancreas	0.045	0.024
Red bone marrow	0.008	0.009
Osteogenic cells	0.011	0.012
Skin	0.006	0.007
Spleen	0.018	0.013
Testes	0.010	0.011
Thymus	0.009	0.010
Thyroid	0.029	0.025
Urinary bladder wall	0.145	0.129
Uterus	0.018	0.014
Total body	0.011	0.011
Effective dose (mSv/MBq)	0.021	0.019
Estimated dose (mSv)	3.0*	3.2**

^*^143 MBq [^18^F]mFBG administered to a 16-year-old female of 68 kg

^**^166 MBq [^18^F]mFBG administered to a 16-year-old female of 85 kg

### Tumour lesion detection

More lesions, however, not statistically significant, were detected on [^18^F]mFBG PET-CT acquired at 1 h post-injection compared to 2 h, with a mean difference of 1.4 (SD 4.4) in SIOPEN score and 0.1 (SD 0.7) in number of soft tissue lesions. To compare lesion detection on [^18^F]mFBG PET-CT with paired [^123^I]mIBG scans, the 1 h post-injection PET-CT was used.

**Fig. 3:**
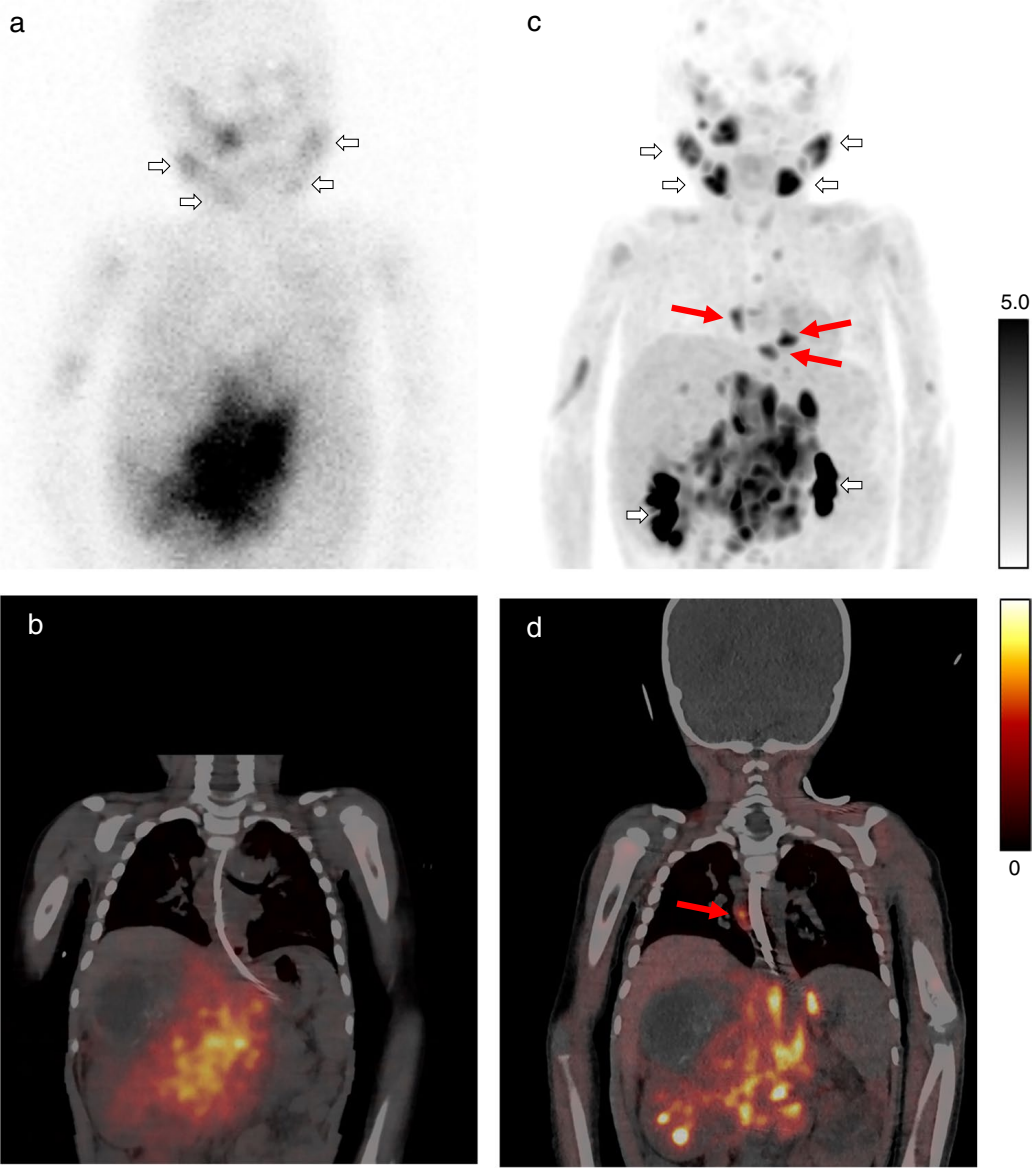
Planar [^123^I]mIBG scintigraphy (**a**) and fused single photon emission computed tomography (SPECT)-CT (**b**) and corresponding [^18^F]mFBG positron emission tomography (PET) maximum intensity projection (MIP) (**c**) and fused PET-CT (**d**) in patient 11 (scan pair 16). The short arrows (**a** and **c**) indicate physiological uptake (salivary glands and pelvicalyceal system), whereas other areas of uptake indicate tumour lesions. The long arrows (**c** and **d**) indicate additional mediastinal lymph node metastases detected on [^18^F]mFBG PET-CT but missed on [^123^I]mIBG scanning. Abbreviations: [^18^F]mFBG = *meta*-[^18^F]fluorobenzylguanidine, [^123^I]mIBG = *meta*-[^123^I]iodobenzylguanidine

**Fig. 4:**
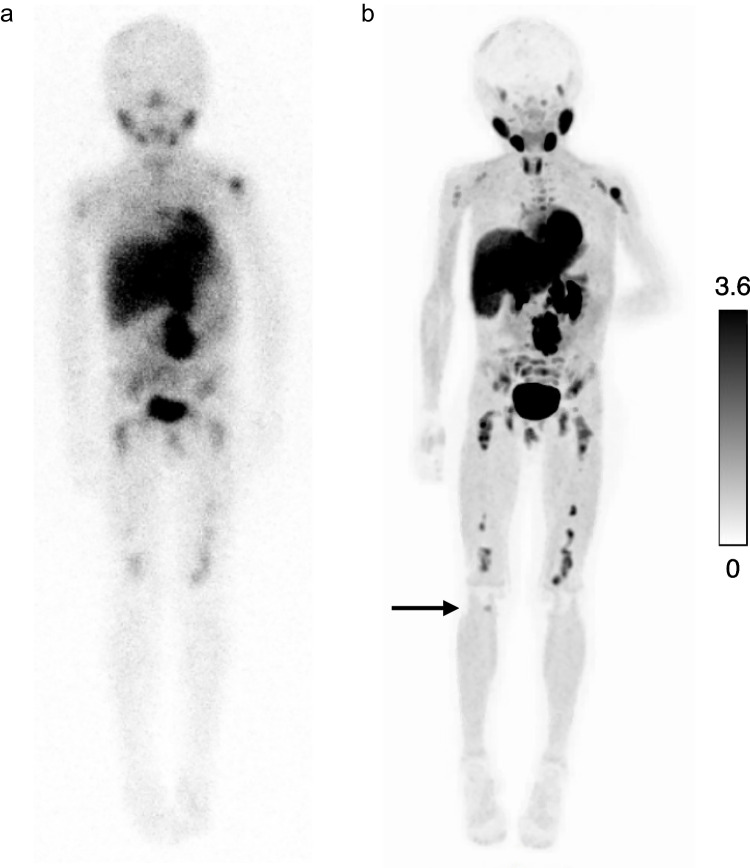
[^123^I]mIBG planar scintigraphy (**a**) and paired [^18^F]mFBG positron emission tomography (PET) maximum intensity projection (MIP) (**b**) in patient 1 (scan pair 1). The arrow indicates a skeletal lesion in the proximal tibia on [^18^F]mFBG PET MIP that was not detected on the paired [^123^I]mIBG scan. This lesion was present on the initial [^123^I]mIBG scan performed at diagnosis. Abbreviations: [^18^F]mFBG = *meta*-[^18^F]fluorobenzylguanidine, [^123^I]mIBG = *meta*-[^123^I]iodobenzylguanidine

Overall, [^18^F]mFBG PET-CT showed similar physiological and pathological distribution to [^123^I]mIBG scanning, however, with higher image resolution, improved tumour lesion delineation, and detection of additional lesions (as seen in the examples in Fig. 3 and Fig. 4). At a patient level, more soft tissue and skeletal lesions were detected on [^18^F]mFBG PET-CT compared to [^123^I]mIBG scanning: on average 2 additional soft tissue lesions and a 6-point higher SIOPEN score. More, equal, and fewer soft tissue lesions on [^18^F]mFBG PET-CT were detected in eight (40%), 11 (55%), and one (5%) of 20 scan pairs, respectively, and a higher, equal, or lower SIOPEN score in 11 (55%), six (30%), and three (15%) of scan pairs, respectively (Table 1). [^18^F]mFBG-positive/[^123^I]mIBG-negative lesions were often present on previous [^123^I]mIBG scans.

At a skeletal segment level, using the SIOPEN skeletal segments, in total 240 segments were scanned. More segments were judged as positive for tumour on [^18^F]mFBG PET-CT (75, 31%) than [^123^I]mIBG scanning (39, 16%). Forty segments were scored as [^18^F]mFBG-positive/[^123^I]mIBG-negative, whereas only four as [^18^F]mFBG-negative/[^123^I]mIBG-positive (example in Fig. S2, Appendix). SIOPEN score per segment on [^18^F]mFBG PET-CT was higher, equal, or lower in 58 (24%), 175 (73%), and 7 (3%) of segments, respectively.

## Discussion

In this prospective pilot study, feasibility of [^18^F]mFBG PET-CT for neuroblastoma imaging was demonstrated by performing 20 paired [^123^I]mIBG and [^18^F]mFBG scans in 14 patients without any adverse reactions. Due to the shorter scan time of [^18^F]mFBG PET-CT compared with standard [^123^I]mIBG scanning, there was a lower procedural sedation rate: 2/20 vs. 10/20, respectively. Compared with [^123^I]mIBG scanning, [^18^F]mFBG PET-CT detected equal or more soft tissue lesions in 95% of scan pairs, and equal or more skeletal lesions in 85% of scan pairs. On average, [^18^F]mFBG PET-CT detected two additional soft tissue lesions and a 6-point higher SIOPEN score per patient.

Frequently, additional lesions were detected on [^18^F]mFBG PET-CT, which is in accordance with the only other study on [^18^F]mFBG PET-CT in five patients with neuroblastoma. Pandit-Taskar and colleagues reported that [^18^F]mFBG PET-CT detected all 22 lesions on [^123^I]mIBG scanning, plus 12 additional lesions [23]. The improved ability to detect lesions with [^18^F]mFBG PET-CT is likely explained by a higher intrinsic spatial resolution and total body view of PET-CT, compared to low resolution scintigraphy and SPECT-CT (with only ~40 cm range). Neuroblastoma imaging with [^18^F]mFBG PET-CT allows for improved tissue delineation and more accurate anatomical localisation of pathological (and physiological) uptake, even near areas showing prominent [^18^F]mFBG activity.

On three [^18^F]mFBG PET-CTs, the SIOPEN score was lower than paired [^123^I]mIBG scanning. In one patient, one solitary new [^123^I]mIBG-positive finding was detected in the right orbital wall, two years after end of therapy, which was not visible on paired [^18^F]mFBG PET-CT or on previous [^123^I]mIBG scans (Appendix, Fig. S2). A probable explanation is that it concerned a false-positive result on [^123^I]mIBG scanning (physiological uptake of the lacrimal gland or eye muscle) due to misalignment of SPECT and CT. In the other two cases, the lower SIOPEN score on [^18^F]mFBG PET-CT did not influence the therapy assessment or clinical management.

In six patients, a follow-up [^18^F]mFBG PET-CT was performed. Similar as for [^123^I]mIBG scanning, SIOPEN scores of the follow-up [^18^F]mFBG PET-CT were lower compared to the previous [^18^F]mFBG PET-CT, which suggests that [^18^F]mFBG PET-CT is suitable for follow-up of treatment response.

[^18^F]mFBG injection seems to be safe and well-tolerated, which is in accordance with previous reports [22–26]. As all patients received less than one microgram of [^18^F]mFBG, pharmacological effects were not expected. Normal distribution of [^18^F]mFBG was almost identical to [^123^I]mIBG with only small differences. The often diffuse pancreatic uptake on [^18^F]mFBG PET-CT was less prominent on [^123^I]mIBG SPECT-CT. This may be related to the more hydrophilic character of [^18^F]mFBG and/or the time point of acquisition because the time-activity curves of the dynamic scans showed a fast decline in pancreatic uptake of [^18^F]mFBG. As determined by 70-min dynamic PET-scans in two 16-year-old females, measured total effective doses for [^18^F]mFBG were lower than for [^123^I]mIBG. Effective doses in these two patients (0.019 and 0.021 MBq/kg) were comparable to the estimation by Pandit-Taskar et al. (0.023 mSv/MBq), estimated in a combined patient cohort of five patients with neuroblastoma (age: 5–23 years) and five patients with pheochromocytoma (age: 16–68 years) [23]. Effective doses of [^18^F]mFBG for different paediatric age categories still need to be determined.

Furthermore, in our relatively young cohort (median age of 4.9 years), there was a reduced rate and length of procedural sedation for [^18^F]mFBG PET-CT compared with standard [^123^I]mIBG scanning because of a shorter scan time. This advantage is especially important for neuroblastoma cases because 90% of patients with neuroblastoma are younger than 5 years of age [1, 2].

To determine the optimal time for imaging, it is important to consider number of detected lesions and tumour-to-background uptake. Pandit-Taskar et al. found that scans > 1 h post-injection showed better tumour-to-background contrast and a higher number of lesions in some patients [23]. Our data show that [^18^F]mFBG uptake in tumour lesions remained stable at 1 h versus 2 h post-injection with only a small decrease in level of background uptake for most organs at 2 h post-injection. Nevertheless, most tumour lesions were detected at 1 h post-injection, however not statistically significant in this small cohort. Taking the shorter waiting time for patients after injection also into account, PET-CT acquisition at 1 h post-injection is preferred over 2 h post-injection.

Two patients were treated with medication known to interfere with [^123^I]mIBG uptake. One patient (patient 13, scan pair 19) was treated with amlodipine (calcium channel blocker) during only the [^18^F]mFBG PET-CT. More lesions were found on the [^18^F]mFBG PET-CT compared to [^123^I]mIBG scanning that was performed prior to the start of the amlodipine. Patient 11 was treated with amlodipine during both [^123^I]mIBG and [^18^F]mFBG scanning (scan pairs 16 and 17). Scan pair 16 had a lower SIOPEN score on [^18^F]mFBG PET-CT compared with [^123^I]mIBG scanning, because the lower arms were scored as positive for skeletal uptake on the [^123^I]mIBG scan, but on the [^18^F]MFBG PET-CT, only prominent muscular uptake was seen without skeletal uptake (Fig. 3).

Some limitations of our study may be considered. The most important limitation is the lack of a “gold” standard, which applies for positive findings on both [^123^I]mIBG scanning and [^18^F]mFBG PET-CT. Histological confirmation of these “lesions” was not feasible due to ethical reasons. In clinical practice, we do regard positive findings on [^123^I]mIBG scanning as neuroblastoma lesions. As [^18^F]mFBG accumulates intracellularly via the same norepinephrine transporter, it is likely to concern real neuroblastoma lesions. With regard to [^18^F]mFBG-positive/[^123^I]mIBG-negative lesions, these were frequently detectable on previous [^123^I]mIBG scans, making it most likely to concern true tumour lesions. Secondly, six patients underwent a second [^18^F]mFBG PET-CT at a later time point, which could have induced selection bias. However, therapy had been given in-between the first and second scan pair, which induces changes in extent of disease. Therefore, follow-up scans of the same patient but at different time points during course of treatment were considered to be independent from the first scan pair. Lastly, the study population was heterogeneous regarding time point of assessment, which could have influenced imaging results. Imaging at diagnosis and earlier in treatment usually shows more disease localizations than at end of treatment.

All in all, the implementation of new PET tracers for the evaluation of neuroblastoma has been slow. [^123^I]/[^131^I]mIBG scanning is well-embedded in neuroblastoma protocols, based on a large body of evidence [34–36]. This is not available for other PET-tracers, such as [^18^F]F-DOPA or [^68^ Ga]Ga-DOTA-peptides. An advantage of benzylguanidine-based tracers, such as [^18^F]mFBG, is that it is taken up via the same NET transporter as [^123^I]/[^131^I]mIBG. This similarity raises hope that the clinical implementation of [^18^F]mFBG will be more rapid.

[^18^F]mFBG PET-CT is a promising alternative to current standard-of-care [^123^I]mIBG scanning in neuroblastoma, which could improve patient care. Sixty percent of patients with high-risk neuroblastoma who are deemed to be in complete remission will eventually relapse. In such cases, [^18^F]mFBG PET-CT could add critical information on the presence of tumour localisations. A follow-up study is underway to confirm these preliminary findings on tumour lesion detection in a larger patient population. Future studies should be aimed at assessing the prognostic relevance of additional lesions on [^18^F]mFBG PET-CT. For [^18^F]mFBG PET-CT, new prognostic scoring systems for quantifying tumour localisations (ideally implementing functional activity) need be established in large clinical trials.

## Conclusion

Results of this pilot study demonstrate feasibility of [^18^F]mFBG PET-CT as new fast, high-resolution imaging technique in neuroblastoma. [^18^F]MFBG PET-CT is more convenient than standard-of-care [^123^I]mIBG scanning, which could lower patient burden for these young children who have to undergo multiple scans. More lesions are detected compared to [^123^I]mIBG scanning, which may contribute greatly to staging and follow-up of patients with neuroblastoma. If future clinical trials confirm results found in this study, [^18^F]mFBG PET-CT is a promising alternative to [^123^I]mIBG scanning that could change standard practice of neuroblastoma imaging.

## Supplementary Information

Below is the link to the electronic supplementary material.

Supplementary file1 (DOCX 1281 KB)

## Data Availability

The datasets generated during and/or analysed during the current study are available from the corresponding author on reasonable request.
