# Correction to: [^18^F]mFBG PET‑CT for detection and localisation of neuroblastoma: a prospective pilot study

**DOI:** 10.1007/s00259-023-06133-3

**Published:** 2023-02-18

**Authors:** Atia Samim, Thomas Blom, Alex J. Poot, Albert D. Windhorst, Marta Fiocco, Nelleke Tolboom, Arthur J. A. T. Braat, Sebastiaan L. Meyer Viol, Rob van Rooij, Max M. van Noesel, Marnix G. E. H. Lam, Godelieve A. M. Tytgat, Bart de Keizer

**Affiliations:** 1 Princess Máxima Centre for Paediatric Oncology, Heidelberglaan 25, 3584 CS Utrecht, Netherlands; 2 Division Imaging & Oncology, University Medical Centre Utrecht, Utrecht, Netherlands; 3 Department of Radiology and Nuclear Medicine, Cancer Centre Amsterdam, Amsterdam University Medical Centres, Amsterdam, Netherlands; 4 Mathematical Institute, Leiden University, Leiden, Netherlands

**Correction to: European Journal of Nuclear Medicine and Molecular Imaging**

https://doi.org/10.1007/s00259-022-06063-6

The article [^18^F]mFBG PET‑CT for detection and localisation of neuroblastoma: a prospective pilot study, written by Atia Samim, Thomas Blom, Alex J. Poot, Albert D. Windhorst, Marta Fiocco, Nelleke Tolboom, Arthur J. A. T. Braat, Sebastiaan L. Meyer Viol, Rob van Rooij, Max M. van Noesel, Marnix G. E. H. Lam, Godelieve A. M. Tytgat, and Bart de Keizer, was originally published Online First without Open Access. After publication in volume 50, issue 4, page 1146 - 1157 the author decided to opt for Open Choice and to make the article an Open Access publication. Therefore, the copyright of the article has been changed to © The Authors 2022 and the article is forthwith distributed under the terms of the Creative Commons Attribution 4.0 International License (http://creativecommons.org/licenses/by/4.0/), which permits use, sharing, adaptation, distribution and reproduction in any medium or format, as long as you give appropriate credit to the original author(s) and the source, provide a link to the Creative Commons licence and indicate if changes were made.

The original article has been corrected.

