# Supplementary material for: [18F]mFBG PET-CT for detection and localisation of neuroblastoma: a prospective pilot study
Source: Eur J Nucl Med Mol Imaging. 2022 Dec 12;50(4):1146–57. doi: 10.1007/s00259-022-06063-6 (PMC9931849; doi:10.1007/s00259-022-06063-6)
Supplement: Supplementary file 1 — Supplementary file1 (DOCX 1281 KB) [file 259_2022_6063_MOESM1_ESM.docx]

**Supplementary Appendix**

**Fig. S1 Images of patient 4 (scan pair 4) who underwent dynamic PET scanning.** PET MIP images acquired at 7 minutes (a), 70 minutes (b), and 120 minutes (c) after [^18^F]mFBG injection. Paired [^123^I]mIBG scintigraphy (d) acquired at 24 hours after [^123^I]mIBG injection

Abbreviations:

[^18^F]mFBG = *meta*-[^18^F]fluorobenzylguanidine, [^123^I]mIBG = *meta*-[^123^I]iodobenzylguanidine, PET = Positron emission tomography, MIP = Maximum intensity projection

**Fig. S2 [^123^I]mIBG-positive/[^18^F]mIBG-negative scan pair (15) performed at the end of therapy.**[^123^I]mIBG SPECT-CT (a) showing uptake of the right orbital wall (arrow) that was not detected on paired [^18^F]mFBG PET-CT (b). The [^123^I]mIBG uptake was not detected on the previous [^123^I]mIBG SPECT-CT performed at diagnosis (c)

Abbreviations:

[^18^F]mFBG = *meta*-[^18^F]fluorobenzylguanidine, [^123^I]mIBG = *meta*-[^123^I]iodobenzylguanidine, PET = Positron emission tomography, SPECT = Single photon emission computed tomography
